# Connection and Protection: A Lifespan Analysis of Technology Use in LGBTQ+ Adults

**DOI:** 10.1093/geroni/igaf122.3906

**Published:** 2025-12-31

**Authors:** Emily Briggs, Jess Francis-Levin

**Affiliations:** University of Michigan, Ann Arbor, Michigan, United States

## Abstract

Finding community and forming social ties looks a lot different today than it did 50 years ago. For members of the LGBTQ+ community, formation of a safe and affirming community adds a layer of complexity to the ever evolving process of developing meaningful social connections. This exploratory study investigates responses to an open-ended prompt in a large multi-measure survey inquiring about technology use in the LGBTQ+ community (N = 338). Participant responses (N = 89) about their technology use provided insight into how social technology is uniquely harnessed by members of the LGBTQ+ community in a sample of young (Age, 18-34, n = 45), midlife (Age, 35-64, n = 31), and older adults (Age, 65+, n = 13). Themes generated from our thematic analysis highlight the different expectations held across the lifespan for community using social technology. While young adults sought out niche spaces tailored to their specific interests, midlife adults discussed using social media to form in person connections, and older adults disclosed details about their in-person community. Furthermore, while older adults expressed the most caution for interacting via online platforms, the theme of using social technology to maintain boundaries with toxic relations was present across all age groups. Taken together, these responses highlight the importance of online spaces as a place for the intentional formation of safe social communities inclusive of the evolving social community needs for adults across the lifespan.

